# Morbidity Burdens Attributable to Various Illnesses and Injuries Among Deployed Active and Reserve Component Service Members of the U.S. Armed Forces, 2023

**Published:** 2024-07-20

**Authors:** Armed Forces Health Surveillance Division Defense Health Agency, US

## Abstract

**What are the new findings?:**

Musculoskeletal disorders in combination with administrative and other health services (ICD-10 “Z” codes) accounted for more than half of all medical encounters in 2023 among service members deployed to the U.S. Central Command (CENTCOM) and Africa Command (AFRICOM). Three common injury conditions occurred among male and female service members deployed to U.S. CENTCOM and U.S. AFRICOM: other back problems, arm and shoulder injuries, and knee injuries.

**What is the impact on readiness and force health protection?:**

Thorough examination of the most common causes of injury and illness during deployment can assist senior leaders in the development and implementation of strategies to reduce preventable medical issues, enhance force readiness, and ensure fighting strength.

## 1 BACKGROUND

Each year, *MSMR* estimates illness and injury-related morbidity and health care burdens on the U.S. Armed Forces and the Military Health System (MHS), and this report updates previous analyses of these burden distributions among active and reserve component service members in deployed settings. While deployed service members are primarily selected from a subset of the active component, the reserve component contributes a substantial portion of U.S. deployed forces.

This report utilizes data from the Theater Medical Data Store (TMDS), which documents service members’ inpatient and outpatient encounters while treated in an operational environment. TMDS receives medical data from Theater Medical Information Program-Joint (TMIP-J) applications, including AHLTA-Theater, TMIP-Composite Health Care System Cache, Mobile Computing Capability, Maritime Medical Modules, and the U.S. Transportation Command Regulating and Command and Control Evacuation System (TRAC2ES).^1^

The health encounters of service members deployed to 2 specific theaters of operation, US Central Command (CENTCOM) and US Africa Command (AFRICOM), are the subject of this report. While U.S. service members are deployed to all the geographic combatant commands, the largest concentrations without access to fixed medical facilities are in the CENTCOM and AFRICOM areas of operation.^2^ While this report focuses on medical encounters of service members treated in CENTCOM and AFRICOM operational environments during the 2023 calendar year, future reports may incorporate other combatant commands as circumstances dictate and data become available.

## 2 METHODS

The surveillance population included all individuals who served in the active or reserve components of the U.S. Army, Navy, Air Force, Marine Corps, or Space Force with health care encounters captured in the TMDS during the surveillance period. Analysis was restricted to encounters where the theater of care specified was CENTCOM or AFRICOM, or where the name of the theater of operation was missing or null; by default, this excluded encounters in the U.S. Northern Command (NORTHCOM), U.S. European Command (EUCOM), U.S. Indo-Pacific Command (INDOPACOM), or U.S. Southern Command (SOUTHCOM) theaters of operations. In addition, TMDS-recorded medical encounters where the data source was identified as Shipboard Automated Medical System, or where the military treatment facility descriptor indicated that care was provided aboard ship, were excluded from this analysis. Encounters from aeromedical staging facilities outside of CENTCOM or AFRICOM were also excluded.

Morbidity burdens attributable to various conditions were estimated by diagnosis distribution according to the 17 traditional categories of the International Classification of Diseases (ICD) system, with an 18th category for COVID-19. Extended ICD-10 (10th Revision) code groupings were also reviewed for the most common diagnoses. The TMDS has not fully transitioned to ICD-10 codes, so some ICD-9 (9th Revision) codes were included. Primary diagnoses that did not correspond to an ICD-9 or ICD-10 code are not reported in this burden analysis.

## 3 RESULTS

A total of 182,943 medical encounters occurred among 53,215 individuals deployed to Southwest Asia, the Middle East, and Africa in 2023. Of those 182,943 total medical encounters documented in 2023 among deployed service members, 84 (0.05%) were recorded as hospitalizations. The majority of medical encounters (n=137,447; 75.1%), individuals affected (n=43,001; 80.8%), and hospitalizations (n=70; 83.3%) occurred among male service members.

In 2023 the largest percentages of medical encounters attributed to a major ICD-10 diagnostic category were coded as musculoskeletal system/connective tissue disorders, followed by administrative and other health services (Z codes; includes factors influencing health status and health service contact) (**Figure**). The most common diagnosis within the musculoskeletal system/connective tissue disorders group was for lower back pain (ICD-10 code beginning with M545) (**Table**).

The percentage of total medical encounters attributed to other health services decreased from 43.5% in 2021 to 25.7% in 2023. COVID-19 accounted for only 0.7% of deployed service members’ total medical encounters in 2023 (**Table**). The percentages of in-theater medical encounters attributed to musculoskeletal system disorders (29.6% to 27.9%) and injuries (7.9% to 7.4%) decreased only slightly from 2019 to 2023 (**Figure**). Lower back pain (M545) was the most frequent ICD-10 diagnostic code for musculoskeletal encounters among both men and women (**Table**). The second-most frequent ICD-10 diagnostic code for musculoskeletal encounters by male service members was pain in the right shoulder (M25511), while for female service members it was pain in the left knee (M25562) (**Table**).

The percentages of in-theater medical encounters attributed to mental health disorders increased from 4.7% to 6.5% during the surveillance period (**Figure**). Adjustment disorder with mixed anxiety and depressed mood (F4323) accounted for the most frequent mental health disorder diagnoses, with a higher percentage of in-theater encounters for this disorder among women (1.4%) than men (0.8%) (**Table**).

## 4 DISCUSSION

As in prior annual reports of illness- and injury-related morbidity and health care burdens in deployed settings, musculoskeletal disorders in combination with administrative and other health services accounted for more than half of the total medical encounters in theater. In prior reports during the surveillance period, encounters for COVID-19 screening contributed to an increase in encounters for administrative and other health services, as this specific Z-code (Z1152) accounted for almost 5% of all in-theater medical encounters in 2022.^3^

This report documents an increased percentage of in-theater medical encounters for mental health disorders, consistent with the 2019-2023 increased rate of in-garrison ambulatory encounters for mental health disorders. The percentage of total ambulatory encounters attributed to mental health disorders in garrison (14.6%) was substantially higher, however, than the percentage observed in theater (6.5%).^4^ No absolute rate comparisons can be made due to the lack of in-theater denominator (person-time) data.

Encounters for certain conditions are generally rare in deployment settings. Some conditions, including diabetes, pregnancy, or congenital anomalies, often preclude service member deployment. Due to medical pre-screening, service members who are deployed demonstrate a lower rate of medical conditions that could interfere with deployment operations than their non-deployed counterparts. Deployed service members are also less likely to require medical care for pre-screened conditions.

When interpreting these results and analyses, several limitations of these data should be considered. Not all medical encounters in theaters of operations are recorded in the TMDS. Some care by in-theater medical personnel occurs at small, remote, or austere forward locations where electronic documentation of diagnosis and treatment is infeasible, and some emergency medical care for stabilization of combat-injured service members prior to evacuation may not be routinely captured in the TMDS. Due to the exigencies of deployment settings that can complicate accurate data reporting or transmission, this report may underestimate the true burden of health care in the areas of operations assessed.

In any review that relies on ICD coding, some diagnosis misclassification should be expected due to coding errors within the electronic health record. Although the aggregated distributions of illnesses and injuries presented in this report are compatible with assessments derived from other examinations of morbidity in military populations (both deployed and nondeployed), instances of highly unlikely diagnostic codes for a deployed population have been observed. This misclassification bias is likely minor and non-differential.

Because this report only includes medical evacuations from CENTCOM and AFRICOM, it does not describe any medical evacuations from the recent deployment of troops to EUCOM, INDOPACOM, and SOUTHCOM. Each area of operation is unique, with vastly different medical assets, medical evacuation capabilities, and deployed service member populations. Consequently, the results from CENTCOM or AFRICOM may not be generalizable to other combatant commands.

## Figures and Tables

**Figure:**
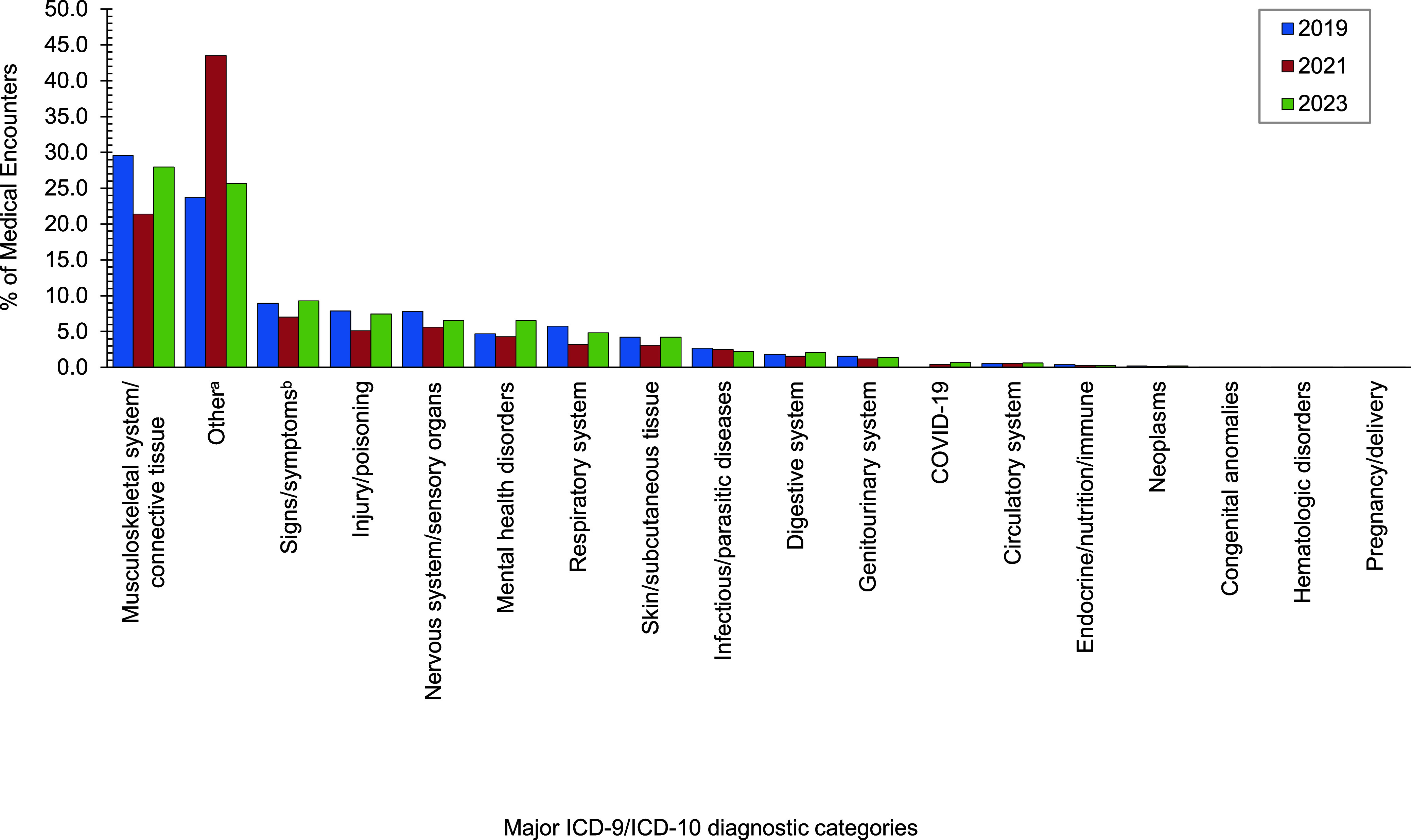
Major ICD-9 and ICD-10 Diagnostic Categories of In-Theater Medical Encounters, Active Component, U.S. Armed Forces, 2019, 2021 and 2023

**Table:** Most Frequent ICD-10 Diagnostic Codes for In-Theater Medical Encounters by Sex, Active Component, U.S. Armed Forces, 2023

		Total		Men				Women			
ICD-10 Code^a^	Description	No.	%	ICD-10 Code	Description	No.	%	ICD-10 Code	Description	No.	%
Z029	Encounter for administrative examinations, unspecified	8,502	4.7	Z029	Encounter for administrative examinations, unspecified	6,208	4.5	Z029	Encounter for administrative examinations, unspecified	2,294	5.0
Z5682	Military deployment status	6,493	3.6	Z5682	Military deployment status	4,919	3.6	Z5682	Military deployment status	1,574	3.5
M545	Low back pain	5,288	2.9	M545	Low back pain	4,364	3.2	Z1159	Encounter for screening for other viral diseases	1,428	3.1
M5450	Low back pain, unspecified	4,766	2.6	M5450	Low back pain, unspecified	3,852	2.8	M545	Low back pain	924	2.0
Z0289	Encounter for other administrative examinations	3,868	2.1	M25511	Pain in right shoulder	3,018	2.2	M5450	Low back pain, unspecified	914	2.0
Z9182	Personal history of military deployment	3,625	2.0	Z0289	Encounter for other administrative examinations	2,960	2.2	Z0289	Encounter for other administrative examinations	908	2.0
Z1159	Encounter for screening for other viral diseases	3,547	1.9	Z9182	Personal history of military deployment	2,948	2.1	J069	Acute upper respiratory infection, unspecified	844	1.9
M25511	Pain in right shoulder	3,538	1.9	M25512	Pain in left shoulder	2,686	2.0	M25562	Pain in left knee	780	1.7
J069	Acute upper respiratory infection, unspecified	3,204	1.8	M25562	Pain in left knee	2,380	1.7	M25561	Pain in right knee	768	1.7
M25562	Pain in left knee	3,160	1.7	M25561	Pain in right knee	2,366	1.7	Z7189	Other specified counseling	687	1.5
M25561	Pain in right knee	3,134	1.7	J069	Acute upper respiratory infection, unspecified	2,360	1.7	Z9182	Personal history of military deployment	677	1.5
M25512	Pain in left shoulder	3,067	1.7	Z1159	Encounter for screening for other viral diseases	2,119	1.5	M542	Cervicalgia	663	1.5
M542	Cervicalgia	2,657	1.5	L731	Pseudofolliculitis barbae	2,110	1.5	F4323	Adjustment disorder with mixed anxiety and depressed mood	632	1.4
R197	Diarrhea, unspecified	2,607	1.4	Z23	Encounter for immunization	2,093	1.5	R197	Diarrhea, unspecified	549	1.2
Z23	Encounter for immunization	2,605	1.4	R197	Diarrhea, unspecified	2,058	1.5	M25511	Pain in right shoulder	520	1.1
Z7189	Other specified counseling	2,465	1.3	M542	Cervicalgia	1,994	1.5	Z23	Encounter for immunization	512	1.1
L731	Pseudofolliculitis barbae	2,112	1.2	Z7189	Other specified counseling	1,778	1.3	Z760	Encounter for issue of repeat prescription	483	1.1
M549	Dorsalgia, unspecified	1,811	1.0	M549	Dorsalgia, unspecified	1,397	1.0	J00	Acute nasopharyngitis [common cold]	432	1.0
J00	Acute nasopharyngitis [common cold]	1,735	0.9	J00	Acute nasopharyngitis [common cold]	1,303	0.9	M549	Dorsalgia, unspecified	414	0.9
F4323	Adjustment disorder with mixed anxiety and depressed mood	1,680	0.9	Z760	Encounter for issue of repeat prescription	1,167	0.8	M25551	Pain in right hip	412	0.9
Z760	Encounter for issue of repeat prescription	1,650	0.9	G4729	Other circadian rhythm sleep disorder	1,065	0.8	F419	Anxiety disorder, unspecified	411	0.9
M25571	Pain in right ankle and joints of right foot	1,325	0.7	F4323	Adjustment disorder with mixed anxiety and depressed mood	1,048	0.8	M25512	Pain in left shoulder	381	0.8
M25572	Pain in left ankle and joints of left foot	1,243	0.7	M25571	Pain in right ankle and joints of right foot	1,000	0.7	M25552	Pain in left hip	358	0.8
U071	COVID-19	1,213	0.7	M25572	Pain in left ankle and joints of left foot	926	0.7	F4320	Adjustment disorder, unspecified	351	0.8
G4729	Other circadian rhythm sleep disorder	1,204	0.7	G4726	Circadian rhythm sleep disorder, shift work type	925	0.7	R21	Rash and other nonspecific skin eruption	330	0.7
R21	Rash and other nonspecific skin eruption	1,195	0.7	U071	COVID-19	920	0.7	Z658	Other specified problems related to pyschosocial circumstances	329	0.7
F4320	Adjustment disorder, unspecified	1,189	0.7	G4700	Insomnia, unspecified	898	0.7	M25571	Pain in right ankle and joints of right foot	325	0.7
G4700	Insomnia, unspecified	1,174	0.6	R21	Rash and other nonspecific skin eruption	865	0.6	J029	Acute pharyngitis, unspecified	318	0.7
F419	Anxiety disorder, unspecified	1,089	0.6	F4320	Adjustment disorder, unspecified	838	0.6	M25572	Pain in left ankle and joints of left foot	317	0.7
M25551	Pain in right hip	1,083	0.6	F419	Anxiety disorder, unspecified	678	0.5	Z733	Stress, not elsewhere classified	315	0.7
G4726	Circadian rhythm sleep disorder, shift work type	1,039	0.6	M546	Pain in thoracic spine	677	0.5	N760	Acute vaginitis	297	0.7
R51	Headache	920	0.5	M25551	Pain in right hip	671	0.5	F4322	Adjustment disorder with anxiety	293	0.6
R519	Headache, unspecified	882	0.5	R51	Headache	636	0.5	U071	COVID-19	293	0.6
M546	Pain in thoracic spine	865	0.5	R519	Headache, unspecified	590	0.4	R519	Headache, unspecified	292	0.6
J029	Acute pharyngitis, unspecified	862	0.5	R109	Unspecified abdominal pain	558	0.4	R109	Unspecified abdominal pain	291	0.6
R109	Unspecified abdominal pain	849	0.5	I10	Essential (primary) hypertension	550	0.4	R51	Headache	284	0.6
M25552	Pain in left hip	844	0.5	M722	Plantar fascial fibromatosis	549	0.4	G4700	Insomnia, unspecified	276	0.6
Z658	Other specified problems related to pyschosocial circumstances	807	0.4	J029	Acute pharyngitis, unspecified	544	0.4	R300	Dysuria	207	0.5
Z733	Stress, not elsewhere classified	805	0.4	R079	Chest pain, unspecified	520	0.4	N390	Urinary tract infection, site not specified	203	0.4
M722	Plantar fascial fibromatosis	733	0.4	Z1152	Encounter for screening for COVID-19	519	0.4	F439	Reaction to severe stress, unspecified	196	0.4
F4322	Adjustment disorder with anxiety	706	0.4	M25569	Pain in unspecified knee	511	0.4	M546	Pain in thoracic spine	188	0.4

Abbreviations: ICD-10, International Classification of Diseases, 10th Revision; ICD-9, International Classification of Diseases, 9th Revision; COVID-19, coronavirus disease 2019.

^a^ Some ICD-9 codes still appear in TMDS. While medical encounters documented with ICD-9 codes were included in the overall analysis for major diagnostic category analysis, the summary of these codes are excluded from this table.
